# Lessons in Innate and Allergic Immunity From Dust Mite Feces and Tick Bites

**DOI:** 10.3389/falgy.2021.692643

**Published:** 2021-06-28

**Authors:** Behnam Keshavarz, Loren D. Erickson, Thomas A. E. Platts-Mills, Jeffrey M. Wilson

**Affiliations:** ^1^Division of Allergy and Immunology, Department of Medicine, University of Virginia, Charlottesville, VA, United States; ^2^Beirne B. Carter Center for Immunology Research and the Department of Microbiology, Immunology and Cancer Biology, University of Virginia School of Medicine, Charlottesville, VA, United States

**Keywords:** allergens, allergic immunity, innate immunity, tick bite, IgE, lone star tick, α-Gal, dust mite

## Abstract

Allergic diseases represent a major cause of morbidity in modern industrialized and developing countries. The origins and development of allergic immune responses have proven difficult to unravel and remain an important scientific objective. House dust mites (HDM) and ticks represent two important causes of allergic disease. Investigations into HDM fecal particles and tick bites have revealed insights which have and will continue to shape our understanding of allergic immunity. In the present review, focus is given to the role of innate immunity in shaping the respective responses to HDM and ticks. The HDM fecal particle represents a rich milieu of molecules that can be recognized by pathogen-recognition receptors of the innate immune system. Factors in tick saliva and/or tissue damage resultant from tick feeding are thought to activate innate immune signaling that promotes allergic pathways. Recent evidence indicates that innate sensing involves not only the direct recognition of allergenic agents/organisms, but also indirect sensing of epithelial barrier disruption. Although fecal particles from HDM and bites from ticks represent two distinct causes of sensitization, both involve a complex array of molecules that contribute to an innate response. Identification of specific molecules will inform our understanding of the mechanisms that contribute to allergic immunity, however the key may lie in the combination of molecules delivered to specific sites in the body.

## Introduction

Allergic immunity consists of a network of cells and mediators that are abundant in the skin and mucosal tissue. The system likely evolved as a rapid-defense mechanism to combat extracellular threats from helminths, ecto-parasites, and venomous animals (1, 2). However, in the industrial world this form of immunity is best known as the cause of allergic diseases, which generally occur as a result of aberrant immune activation to otherwise innocuous environmental antigens. Contemporary understanding of allergic immunity includes not only IgE but also a variety of other cells and mediators that include Th2 cells, ILC2 cells, mast cells, eosinophils, and basophils. Because Th2 cells play a critical role, allergic immunity is also often referred to as Th2-related, or type-2 (3). Despite the fact that it has been over 50 years since IgE was characterized, there remain many gaps in our understanding of the mechanisms and pathways that contribute to allergic immunity. Nonetheless, it is increasingly clear that the evolutionarily ancient innate immune system must play a critical role in shaping the development of allergic responses (4). There are two main ways that activation of the innate immune system could contribute to allergic disease. Firstly, agents which are recognized by the innate immune system could contribute to allergic symptoms via mechanisms that are independent of IgE antibodies. Secondly, innate immune pathways can provide inflammatory cues that promote IgE sensitization and/or maintenance of IgE. To frame this discussion about the role of innate immunity in allergic disease, here we focus on two taxonomically-related arthropods that have strong associations with allergic disease (5). Specifically, we discuss the relevance of house dust mite (HDM) feces and tick bites as discrete entities that are well established triggers of IgE responses. Our emphasis is on studies carried out in humans or with human samples, but we also touch on some animal studies that provide additional support.

## Dust Mites

### Early Insights Into Dust Mite Allergy and the Relevance of Particles

When house dust mites were first established as a major source of house dust allergens, which occurred around 1970 as children in the Western World were spending more and more time indoors, purification of the protein allergens from those acarids became an important objective. Many allergens were identified by cross radio immunoelectrophoresis. Those included “allergen 42,” which Henig Lowenstein recognized as an important allergen (6). In 1978, Chapman purified *Dermatophagoides pteronysinnus* allergen 1 (Der p 1) and that rapidly led to the ability to measure the protein and to the recognition that the mite fecal particles were the major source of allergen that accumulated in cultures (7, 8). Work by Tovey (9) went on to establish that the fecal particle is the major form in which mite allergens become airborne in houses. Further, the size of those particles at 20–35 microns in diameter is such that they contain sufficient allergen for a single particle to induce a positive prick test in an allergic individual. Ultimately these particles turned out to be a treasure trove of different proteins and other molecules that could contribute to the immune response to an increasing list of fully defined protein allergens (see www.allergen.org). In many cases the allergens themselves had intrinsic capacity to activate innate immune pathways, though there were also many other biologically active molecules. These molecules included mite DNA, bacterial DNA, chitin, β-glucan and others (10, 11). Although important concepts and nomenclature in innate immunity had yet to be coined and described by the likes of Charles Janeway Jr., Ruslan Medzhitov, Bruce Beutler, and others, it is now clear that structurally diverse molecules present in HDM are recognized as “Pathogen-associated molecular patterns” (PAMPs) or “Danger-associated molecular patterns” (DAMPs) by the germline-encoded innate immune system (4, 12–14). This is facilitated by several families of pathogen–recognition receptors (PRRs) of which the Toll-like receptor (TLR) family is perhaps best known.

**Figure 1:**
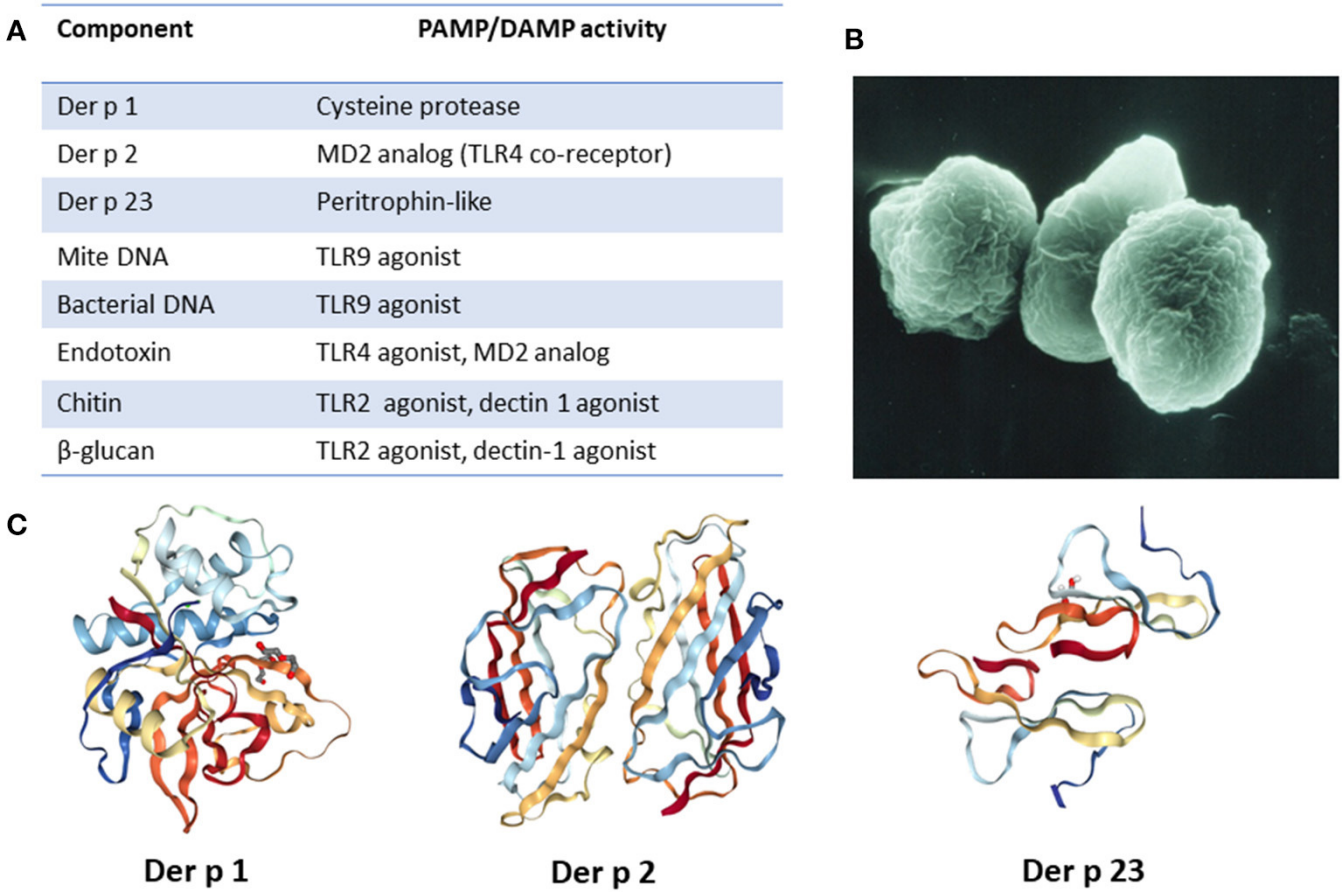
Major HDM allergens and associated constituents present in fecal particles. **(A)** List of relevant allergens and molecules and their PAMP/DAMP activity. **(B)** Image of fecal particle (image courtesy of Euan Tovey). **(C)** Molecular structure of major HDM allergens. Images created with the PDB ID and associated publication, NGL Viewer (16), and RCSB PDB.

Considering the size and known contents of the mite fecal particles, it is interesting to think about the published evidence on delivery of innate receptor ligands and their efficacy as adjuvants. Medzhitov et al. (15) at Yale found clear evidence that the ligands need to be physically “close” to the relevant antigen. Thus, there is every reason to think that one or more PAMPs (or DAMPs) within the fecal particle are relevant to the immune response to the allergens (Figure 1). Taken together, mite allergens present an example where the activators of several innate pathways are inevitably physically close to the mite allergens. Equally, we would argue that the combination of the many different components in mite feces, in addition to the abundance of HDM allergen in dust, is what makes dust mite such a “good” allergen (17). In some countries the dominance of mite allergens in relation to asthma has been truly remarkable. Typical examples include the UK and Japan in the 1980's, New Zealand in the 1990's and Australia in 2000 (18–22). As late as 2012, Heymann studied children presenting to hospital with acute asthma in San Jose, Costa Rica. Those studies demonstrated that IgE to mite allergens was not only highly prevalent, but also much higher titer than those to other allergens in sera from asthmatic patients (23). In that study, the combination of high titer IgE to mite and a positive test for rhinovirus was very strongly associated with acute asthma (odds ratio > 30). We would also emphasize that in an environment with very high levels of exposure to both HDM and cat (e.g., New Zealand), mite allergens are far more important allergens in relation to asthma than cat allergens are (20, 22).

### Allergens and Other Molecules in HDM That Trigger Innate Immunity

During the 1970's and 1980's, dust mite allergens were extensively purified and characterized (6, 7, 24). Up to the late 1980's it was assumed that the IgE response to these allergens was facilitated by direct allergen recognition and activation of Th2 cells. Prior to this time there was little awareness of innate immunity and of its importance in acting as a bridge to T cell activation (25, 26). We are now aware of more than 30 IgE-binding HDM allergens, many of which have a range of functional biological activities and can directly or indirectly trigger innate immune responses (27, 28). Among these, Der p 1, Der p 2, and Der p 23 are identified as the major allergens of *D. pteronysinnus* (29).Of note, these correspond to group 1, group 2, and group 23 allergens of other species of HDM. Consistent with a prior report, we have shown that among children with asthma that the most important allergens are in the order of Der p 1 = Der p 2 > Der p 23 (30, 31). Further, the quantities of IgE to these three components added together can be equivalent to 50–80% of the amount of IgE to crude dust mite allergen extract. Der p 1 was initially recognized as a serine protease and was demonstrated to have the ability to cleave tight junctions, open up epithelial barriers and remove surface receptors such as CD25 and CD23 (7, 25, 26). Disruption of epithelium increases allergen uptake by dendritic cells in the mucosa and contributes to tissue damage. Epithelial disruption by Der p 1 also leads to production of extracellular “alarmin” molecules, such as ATP, that have DAMP activity (32). In turn, ATP can promote release of IL-33 from the epithelium. By interacting with its cognate receptor ST2 that is expressed on Th2, ILC2, mast cells and basophils, IL-33 is increasingly recognized as an important early mediator of type-2 immune responses (33, 34). Of note, emerging evidence suggests that IL-33 can also act directly as a sensor for Der p 1 and other allergen proteases (35). The second major allergen purified, Der p 2, has structural and biochemical similarity with the TLR4 co-receptor MD-2 (17, 24, 35–37). As such, it may enhance lipopolysaccharide (LPS) effects on TLR4, thereby promoting production of pro-inflammatory cytokines. Der p 23 is a peritrophin-like protein that was recently acknowledged as a third major allergen (30, 38). It is speculated to have chitin-binding properties, as has been reported for peritrophin, though this has yet to be experimentally confirmed (39, 40). Because Der p 23 is only present in mite fecal particles in minimal quantities it raises some confusion, but it is nonetheless able to induce high titer IgE antibodies in a large proportion of subjects. Several other HDM allergens have also been shown or are thought to be able to activate the innate immune system. For example, Der p 3, Der p 6 and Der p 9 all have protease activity (41). In addition, Der p 5, Der p 7, and Der p 21 share the feature of being lipid-binding proteins (27). Interestingly, a number of important allergens in plants, mammals, and arthropods have been found to be lipid-associated and it is thought that bound lipids can contribute to innate signaling by interacting with TLRs (42). Consistent with this view, Der p 5 has been demonstrated to activate respiratory epithelial cells in a TLR2-dependent manner (43).

As previously mentioned, HDM fecal particles are a rich carrier of not only allergenic proteins but also other components that include mite DNA, bacterial DNA (44), chitin, environmental pollutants, microorganisms (fungi, bacteria, virus, etc.) and microbial compounds (e.g., endotoxin), all of which could act as PAMPs or DAMPs (45). Chitin is a polysaccharide that is abundant in HDM exoskeleton but also found as a component of fecal particles (46). Signaling via TLR2, chitin has been shown to promote Th2 sensitization (47). Another PAMP present in HDM fecal particles (possibly from fungal sources), which has been associated with allergic inflammation is the polysaccharide β-glucan. There have been some conflicting reports on the downstream signaling that is triggered by β-glucan, with putative receptors being TLR2, dectin-1, or complement receptors (48–50). TLR4 activation by bacterial endotoxin present in HDM extracts has also been reported (48, 51). A detailed understanding of the molecular details of how HDM interact with the innate immune system has major challenges. As outlined by Jacquet (52), the exact composition of house dust is not clearly known and there can be major variations in allergen composition in different dust samples. It is clear, however, that HDM fecal particles are a rich source of allergens and other molecules which have PAMP/DAMP activity. It is likely that the abundance and diversity of such molecules is a major factor that contributes to HDM allergenicity.

## Tick Bites

Our interest in the relevance of tick bites to allergic disease stems directly from work on a form of delayed allergy to mammalian meat caused by IgE antibodies to the oligosaccharide galactose-α-1, 3-galactose (α-Gal) (53). Because the allergy can manifest after exposure to products other than meat, such as dairy and gelatin, and also certain biologics derived from mammalian cell lines, the allergy is now best known as the α-Gal syndrome (AGS) (54, 55). Work over the past decade has clearly established that ticks, which are obligate blood-feeding ectoparasites, are the dominant cause of IgE sensitization to α-Gal. As a consequence there are reasons to think that α-Gal is a good model for studying both the relevance of tick saliva and the role of the skin in the origins of allergic immunity (56).

### Tick Bites and IgE to Galactose-α-1,3-Galactose (α-Gal)

It is possible that the innate biological activity of α-Gal has been studied since the original observation that rabbit red blood cells could activate the alternate pathway of human complement leading to red blood cell lysis (57). It had already been pointed out by Landsteiner and Miller (58) in 1925 that rabbit red cells carried a “B like” antigen that had many features in common with B blood group substance. The full structure of α-Gal and appreciation of the “natural” IgG antibody responses to this epitope were later studied by Galili (59). He pointed out that rabbit red cells had particularly high levels of α-Gal on their surface, and also proposed an evolutionary mechanism to explain the 100% loss of α-Gal in higher primates (60, 61). This mechanism proposes that ~25 million years ago a modest or small percentage of the primate population had loss-of-function mutations in the α-1, 3-galactosyltransferase required to synthesize the primate α-Gal glycan. Members of this small group no longer synthesized α-Gal and as a consequence were able to make antibodies that could recognize this oligosaccharide expressed on the surface of human pathogens. The hypothesis also includes a highly fatal pandemic caused by a virus or bacterium which carried this epitope. The *coup de grâce* in this “catastrophic selection” is that those individuals who produced IgG antibodies to α-Gal had a selective advantage (61). Only those who had pre-existing anti-α-Gal antibodies survived. Our own interest in α-Gal arose as a consequence of an investigation into allergic reactions that were occurring upon the first infusion of the monoclonal antibody (mAb) cetuximab in patients with carcinoma of the colon (62). A factor creating special interest was that these reactions were most common in an area focused on Tennessee, Oklahoma, North Carolina, Arkansas, and Virginia. Investigation of pretreatment sera from patients treated with cetuximab in Tennessee established that the reactions were almost exclusively in patients with pre-existing IgE antibodies specific for α-Gal epitopes that were expressed on this mAb (63). Following up on the regional connection, we realized that this was similar to the reported map of maximum incidence of Rocky Mountain Spotted Fever (RMSF) and best explained by the prevalence of a tick which is primarily carried by deer which have been allowed to infest the suburban areas of the Eastern United States (64, 65). It then became clear that this form of allergic disease was also present in several other parts of the world, including Australia, Sweden, France, Germany, and Japan (66). The striking feature from our point of view was that in each of these countries it appeared that the cause of sensitization was tick bites.

The question that is posed is why should IgE sensitization to the α-Gal epitope be so strongly associated with tick bites? Relatedly, why do other forms of α-Gal exposure, which are sufficient to lead to robust IgM, IgG, and IgA against the glycan in almost all healthy humans, not lead to α-Gal sIgE (67, 68)? It is important to recognize that humans are routinely exposed to bacteria that express α-Gal which are part of the normal gut flora (69, 70). In addition, mammalian meat (e.g., beef, pork, and lamb) and/or dairy, products which all express α-Gal, are also routinely consumed in a Western diet (54). Even so, α-Gal sensitization has been uncommon in several population-based studies in areas where tick bites are rare or absent. For example, neither the syndrome nor IgE antibodies to α-Gal have been reported at appreciable levels in Northern Sweden or the mountain states in the Western USA (71–73). There may well be two important parts to these questions: (i) tick bites occur through the skin, whereas “normal” exposure to α-Gal occurs via the gut and (ii) constituents present in tick saliva are recognized by the innate immune system and act as robust type-2-promoting adjuvants. It is important to recognize that α-Gal itself can be considered to have intrinsic PAMP activity given that it is the target of pre-existing IgM, IgG, and IgA antibodies. In addition, α-Gal can be recognized by galectin-3, a member of the lectin family with carbohydrate binding domains (67, 74).

### Tick Bites as a Model for Understanding Allergic Immunity

**Figure 2:**
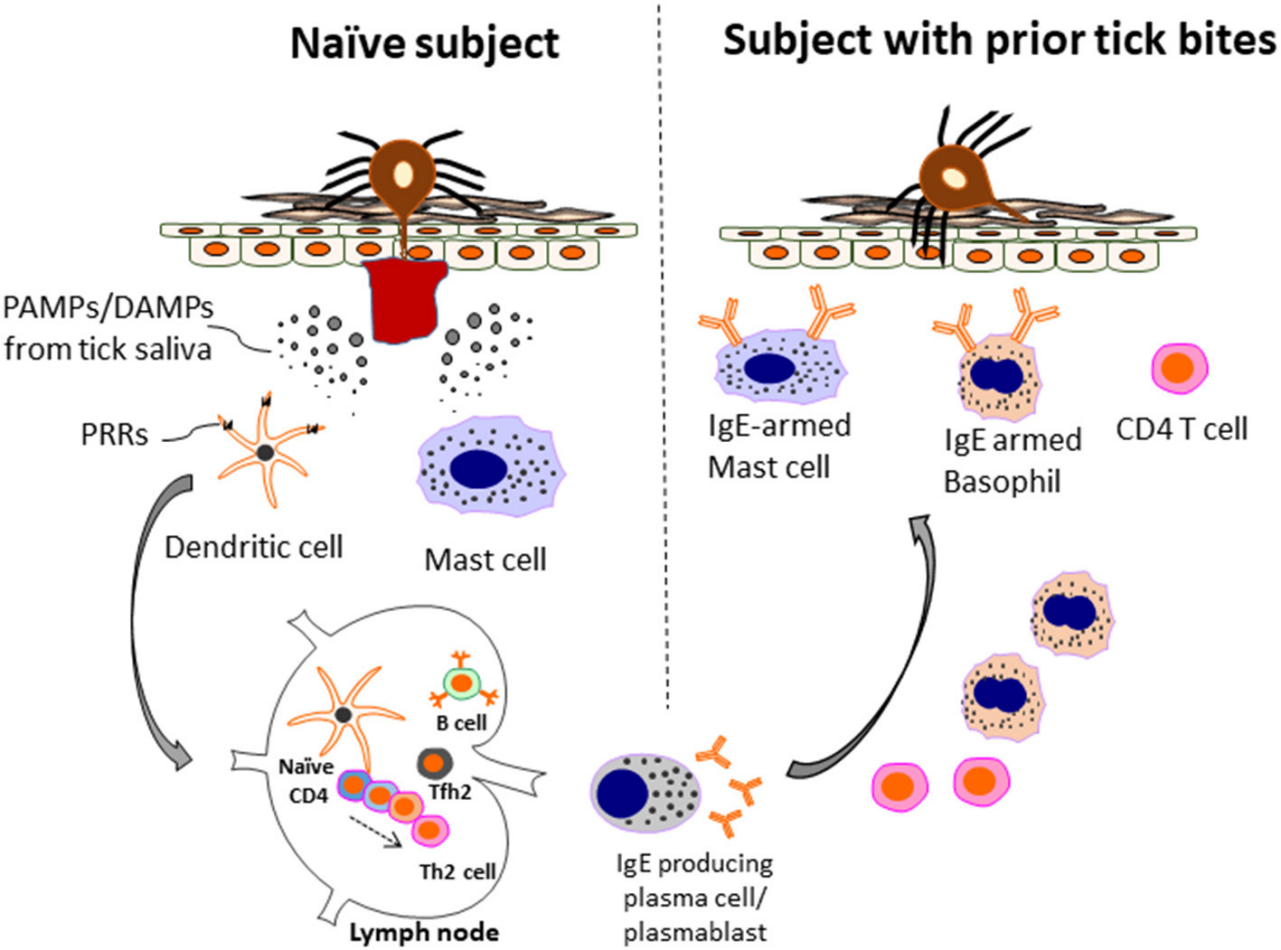
Model of allergic immunity in anti-tick defense. A host without prior tick bites (naïve subject) lacks protective immunity and can have a prolonged tick feed. However, during this initial exposure Th2 cells and IgE-producing B cells are generated. The mechanism includes recognition of PAMPs/DAMPs present in tick saliva by PRRs (e.g., TLR4 and others) present on innate immune cells, such as dendritic cells. In turn, anti-tick IgE binds to mast cells, and basophils *via* the FcεR1 receptor expressed selectively by these cells. Th2 cells and basophils are recruited to the skin. IgE-armed basophils play a particularly important role in acquired tick defense (ATR) to subsequent tick bites. The amount of IgE and number of basophils can be enhanced after repetitive tick bites. As a consequence, both tick feeding and pathogen transmission are impaired. The anti-tick response can include IgE to *a*-Gal, but also other tick antigens that have yet to be characterized. Adapted by permission from Springer Nature, Current Allergy and Asthma Reports, Galactose-α-1,3-galactose: Atypical food allergen or model IgE hypersensitivity; Wilson et al. (56).

In the United States, the primary cause of tick bites in the area where the disease was first recognized is the lone star tick, *Amblyomma americanum* (64). In other countries the ticks that are recognized as being relevant are generally the most common species biting humans. This may be relevant in the sense that sensitization to the level where the syndrome becomes clinically obvious may require repeated tick bites (75–77). It is also possible that the length of tick mouthparts are relevant to sensitization (or allergic reactions to ticks), as ticks such as *A. americanum* and *Ixodes holocyclus*, which are highly associated with α-Gal sensitization, have relatively longer mouthparts than other tick species (78). The connection between α-Gal sensitization and tick bites has also been bolstered by recent studies showing that the α-Gal glycan is expressed in salivary glands of several species of ticks (79–82). Importantly, ticks in which α-Gal have been identified include the major ticks associated with α-Gal sensitization in the USA (*A. americanum*) and in Europe (*I. ricinus*). In the United States there are large areas of the country where Lyme disease, which is transmitted by *l. scapularis*, is common. Despite the fact that *I. scapularis* can also express α-Gal (79, 83), this tick does not appear to be a significant cause of sensitization to α-Gal. The basis for this observation is that areas of the northeast and upper Midwest have few α-Gal cases (71, 73). Interestingly, the tick bites we studied between 2007 and 2014 had a striking characteristic which was intense itching after the bites. In Central Virginia, a positive answer to the question “have you experienced itching after a bite that lasted more than 2 weeks” was very strongly associated with an IgE response to α-Gal approaching an odds ratio of 10 (*P* < 0.001) (71). By contrast, a major study on Lyme disease was carried out on Block Island which lies 15 km off Rhode Island (84). In that study ~1,500 inhabitants were questioned annually for 10 years about tick bites including the question “Have you experienced itching after a tick bite in the last year.” Those subjects who reported itching after a bite on three or more occasions were 82% *less* likely to have positive serology to Lyme disease antigens (84). We consider that there are two important messages from these studies of pruritic tick bites. The first is that itching after a tick bite is probably a negative characteristic relative to the transmission of *Borrelia burgdorferi* and other tick-borne pathogens. It can be assumed that itching at the site of a tick bite occurs because of a pre-existing Th2-related immune response, and supports the view that in most cases sensitization to α-Gal (and other tick antigens) requires repeated bites (75, 84). The second message is the suggestion that allergic immunity, which involves the elaboration of histamine and other mediators that contribute to pruritis, is an adaptive response that evolved in mammals, in part, to defend against ticks and the pathogens they harbor (see Figure 2) (1, 2, 85). This message is important because historically some have viewed Th2-related immunity as an anti-inflammatory response that is maladaptive and directed primarily by and for the advantage of the tick (86, 87). Part of the confusion about the teleology of type 2 immunity and ticks probably relates to earlier views that suggested that Th1 and Th2 responses represented binary arms of the immune system. However, models of CD4 differentiation that suggested that Th2 immunity reflected an “anti-inflammatory” response have largely fallen out of favor with more nuanced views of CD4 T cell heterogeneity (86–88).

Animal models have also played important roles in informing our understanding of the role of allergic immunity in defense against tick feeding. As early as the 1930's work on guinea pigs revealed that immunity was acquired following a primary tick infestation that provided protection against a subsequent tick infestation(s) (89). As recently reviewed by Karasuyama et al. (78), work over the ensuing decades has revealed important roles for Th2-related cells and mediators—namely basophils, eosinophils, mast cells, and IgE—in conferring this protection. Studies suggest that basophils play a particularly important role in anti-tick defense. First, basophils accumulate at tick bite lesions, both in humans and in animal models, with increasing frequency with serial tick bites (53, 90–92). Second, ablation of basophils with antibodies or with genetic approaches in animal models leads to a decrease in acquired immunity (93, 94). Thus, basophils (and/or mast cells) that become armed with anti-tick IgE following a primary tick exposure likely contribute to acquired resistance to future tick bites and explain the presence of pruritis in individuals who experience frequent tick bites. Further evidence for this model is the fact that: (i) tick saliva contains several histamine-binding proteins which act to block the activity of host-derived histamine (78) and (ii) administration of an H1 histamine blocker to mice was sufficient to impair acquired resistance to a 2nd tick infestation (95). Detailed investigation revealed that the major source of the histamine at the site of the tick lesion was from basophils, not mast cells (95). A recent study introduced extracts from pathogen-free *A. americanum* larvae subcutaneously into C57BL/6 mice. Interestingly, there was a marked increase in total serum IgE following sensitization at day 0 and 7, which was further pronounced with a challenge at day 31 (96). This extract (which did not have detectable α-Gal in it) did not induce IgE to α-Gal, but did induce IgE specific to the tick extract. However, when exogenous α-Gal glycoprotein was spiked into the larval extract, IgE specific for α-Gal was detected in the mouse serum. To investigate elements of innate immunity that could be priming the IgE response, a TLR screen was conducted. The screen revealed that the tick extract had robust TLR2, TLR4, and TLR 5 activity. They additionally showed that the response was MyD88 dependent, with a specific B-cell intrinsic role for MyD88 in promoting IgE class switch (96). The specific factors that acted in this model *via* TLR/MyD88 pathways to promote IgE have yet to be elaborated.

### What Are the Factors in Tick Saliva That Contribute to Allergic Immunity?

Tick saliva contains a large array of biologically active molecules that are dynamically regulated during a blood meal. These factors play a critical role in facilitating a successful blood feed by promoting tick attachment, impairing host hemostasis, and modulating host immunity. The reader is referred to a recent review by Patricia Nuttall which has an excellent overview of the topic (97). Interestingly, Alejandro Cabezas-Cruz and James Valdés asked whether ticks should be considered as venomous ectoparasites. This was based on the observation that several of the protein families identified in tick saliva have close homologs in the venom of snakes, scorpions, and other venomous animals (98). The relevant proteins include defensins, lectins, lipocalins, Kunitz-like peptides, metalloproteases, and phospholipase A2. Non-protein factors, including prostaglandins, nucleosides, endocannabinoids, and microRNA are also present in tick saliva (97). When considering the role of tick saliva on host immunity one must consider that there are factors in tick saliva that act to impair the host immune response and also factors that are recognized by the innate immune system which prime type 2 immunity. This dynamic interplay between tick and host has been described by some as an “immunologic arms race” (99). Examples of tick salivary factors that can impair the host response include histamine-binding proteins, the purine nucleoside adenosine, and the protein Salp15, which is produced by *I. scapularis* and been shown to impair CD4 T cell proliferation (87). However, the question most germane to the current discussion is: what are the factors that are recognized by the host immune system in the skin that contribute to IgE induction? Clearly there must be potent Th2-promoting PAMP/DAMPs present in tick saliva, but at this point the role of specific factors is not well characterized. Examples of molecules that have been proposed or could be relevant in driving IgE class switch include prostaglandin E2 (100) and/or phospholipase A2 (PLA2) (101). PLA2 is an intriguing possibility as it is: (i) found in abundance in the saliva of *A. americanum*, (ii) honey bee PLA2 is the major allergen of bee venom, and (iii) honey bee PLA2 has intrinsic PAMP activity (101–103). Given the complexity of tick saliva it would not be surprising if other PAMPs/DAMPs with Th2-promoting activity, including those that are lipid-based, are discovered in the future. It is also important to consider that there are communities of bacteria, viruses and eukaryotes present in tick saliva that could act as PAMPs (104). Finally, host factors that are elicited indirectly as a consequence of damaged epithelium, such as IL-33 or ATP, also warrant further investigation (32, 35).

## Conclusion

**Figure 3:**
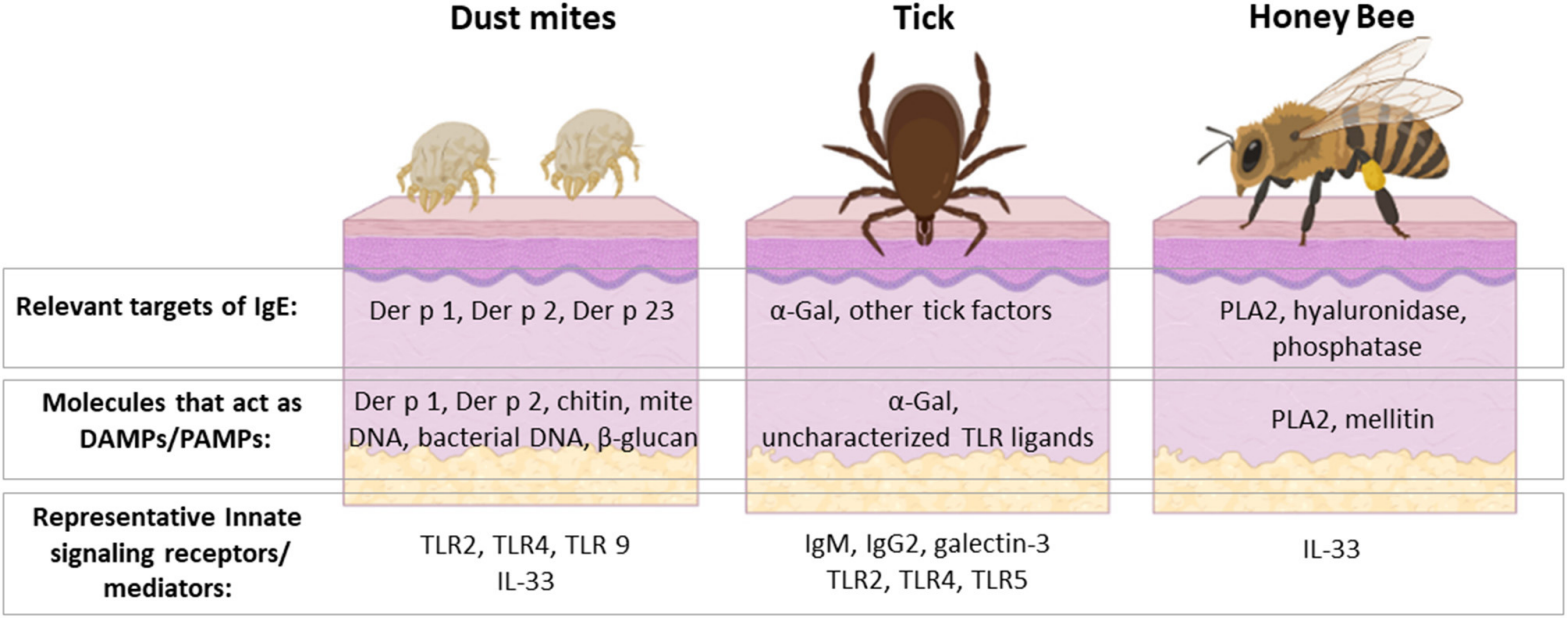
HDM, ticks and venomous insects are important causes of allergic disease with several shared features but also important differences. They are all members of the Arthropod phylum and can interact with the host at the skin barrier, though HDM sensitization occurs dominantly via the respiratory mucosa. The major allergens of HDM, ticks and honey bee are unique from each other, but share the distinction of acting to stimulate host innate immunity. Some allergens, such as Der p 2 and α-Gal, are recognized directly by innate immune receptors. Other allergens, such as Der p 1 and PLA 2, have enzymatic activity that leads to activation of innate mediators, including IL-33. Activation of these innate pathways is incompletely understood but leads to induction of Th2 cells and IgE class-switch. Rhinitis, asthma, and anaphylaxis are allergic sequelae that can occur upon re-exposure to the relevant arthropod. On the other hand, these symptoms are manifestations relating to allergic host defenses that have evolved to defend against these organisms. Created with BioRender.com.

The scientific community has made great strides over the past few decades in unraveling the pathways that contribute to IgE sensitization. Research increasingly suggests that allergic immunity evolved as a rapid defense mechanism to combat helminths, ecto-parasites, and venomous animals. This hypothesis is supported by significant gains in our knowledge of the innate immune system and also by insights into allergic immune responses elicited by two acarid arthropods with very different modes of interaction with the host surface. Allergic sensitization to HDM fecal particles occurs dominantly via the respiratory mucosa, whereas tick bites promote IgE sensitization through the skin. Work on dust mites in the 1970's provided early clues about the importance of particles in allergen sensitization. It also hinted that biologically complex molecules, such as those in mite feces, would be relevant to priming an allergic response. More recent work suggests that proteolytic disruption of the epithelial barrier is an indirect mechanism whereby HDM and other environmental allergens drive innate pathways. Our understanding of the factors in tick saliva that contribute to IgE class-switch and Th2-priming remain in their infancy, but there are good reasons to think that tick saliva is a rich source of molecules with such biological activity. The robust allergic response that occurs to both tick bites and HDM fecal particles is likely explained by conserved features, including: (i) the delivery of multiple PAMPs/DAMPs into a local area of the skin or mucosa and (ii) effects which disrupt the epithelial barrier. The latter is clearly consistent with the epithelial barrier hypothesis of allergic disease, as recently summarized by Cezmi Akdis (105). Work to clarify the specific factors that contribute to innate activation is ongoing, but has been hampered by a reliance on the use of animal models and cell-based co-culture systems. Emerging tools and technologies can be expected to play an important role in promoting further advances. Ongoing investigations into ticks, mites and also venomous insects have the potential to provide more information about the origins and development of allergic immunity (Figure 3).

## Author Contributions

BK, LE, TP-M, and JW contributed to the writing and graphics of the manuscript. All authors contributed to the article and approved the submitted version.

## Conflict of Interest

The authors declare that the research was conducted in the absence of any commercial or financial relationships that could be construed as a potential conflict of interest.
